# Self-arranged exposure for overcoming blood-injection-injury Phobia: a case study

**DOI:** 10.1080/21642850.2014.916219

**Published:** 2014-05-21

**Authors:** Michelle R. Pitkin, John M. Malouff

**Affiliations:** ^a^ Psychology, University of New England, Armidale, Australia

**Keywords:** blood, injection, injury, phobia, self-exposure, treatment

## Abstract

Blood-injection-injury (BII) phobia is both common and dangerous, because it can lead to avoidance of medical procedures for diagnosis and treatment. It also tends to prevent individuals from donating blood for use in the healthcare of others. BII phobia often has an unusual characteristic for a type of phobia – fainting. The typical treatment for BII phobia involves teaching the client how to avoid fainting and staging multiple gradual-exposure trials for the client. In this case report, an adult with the phobia obtained initial, mostly written, guidance from a psychologist, arranged her own applied muscle-tension practice sessions to learn how to keep from fainting, created her own fear hierarchy, and staged exposure trials herself, ending years of avoidance of blood withdrawal. By the end of the trials, she was able to give blood for a medical test and to donate blood for the first time in her life and to work as a volunteer at a blood-donation center. The results provide the first evidence that adults with BII phobia can end the phobia by arranging their own sessions of applied-tension practice and gradual self-exposure. The results suggest a new option for treating specific phobias in general with some adults: initial professional guidance followed by self-arranged gradual-exposure trials.

## 1. Background

Blood-injection-injury (BII) phobia is an acute fear and avoidance of stimuli or invasive medical procedures associated with blood, injections, and injury (Lillecreutz, Josefsson, & Sydsjö, 2010). BII phobia is relatively common; 3–4% of adults have the disorder at some time in their lives (Bienvenu & Eaton, 1989). This type of phobia is unusual in that individuals with it often faint, due to a vasovagal reflex (Ost & Sterner, 1987). The fainting can be dangerous, sometimes leading to injuries from falling and striking one's head. This phobia also can have dangerous consequences when it leads to avoidance of needed medical tests and procedures and when it leads to inability to function in situations involving injury to oneself or to others. The phobia can also limit occupational choice by eliminating employment in medical and similar settings and can restrict life choices, such as traveling to countries for which vaccinations are recommended. Furthermore, the phobia often prevents individuals from donating blood that is crucial to the medical treatment of others.

The usual, relatively effective treatment for specific phobias involves a mental health professional providing the phobic person with gradual exposure to the feared stimuli (Wolitzky-Taylor, Horowitz, Powers, & Telch, 2008). That treatment often requires several sessions with a suitably trained psychologist. For blood phobia, the expert typically first helps the client learn applied muscle tension to prevent fainting and then stages the exposure trials (Ayala, Meuret, & Ritz, 2009; Chapman & DeLapp, 2013; Holly, Torbit, & Ditto, 2012; Mednick & Claar, 2012; Ost & Sterner, 1987). What is unknown is whether an individual can overcome blood phobia through self-arranged muscle-tension practice and exposure trials. Arranging these sessions without the presence of a professional has the potential value of making the treatment available to many individuals who would not or could not find a local, qualified, affordable professional to arrange the sessions for them. The purpose of this paper is to provide a demonstration of the feasibility of effective BII treatment with minimal therapist involvement.

## 2. Method

### 2.1. Participant

The individual with a BII phobia was one of us (Pitkin; P), a 35-year-old female undergraduate psychology student who had had a phobia of giving blood via venipuncture for approximately 20 years. The severity of the phobia resulted in either fainting or feeling faint during attempts to give blood for medical tests and feeling faint when she accidentally cut herself, followed by avoidance of medical health check-ups if she believed they would require a blood sample. Before the intervention, her two most recent blood sample tests were taken four years prior for an illness that required hospitalization for one week. She experienced extreme anxiety and near-fainting and subsequently refused to give a third blood sample that was necessary for further medical analyses. Before the start of treatment, P felt ill just seeing a sign for a blood-donation center. She wanted to overcome the phobia in order to eliminate her fear and avoidance of blood withdrawal and to be able to donate blood for use by others.

### 2.2. Intervention

P began work on overcoming her phobia as part of a self-change project for a course on behavior modification. She continued with the project after the course ended. One of us (M) provided P with an article (Ost & Sterner, 1987) on applied muscle tension for preventing fainting. Applied muscle tension involves tensing the arms, legs, and torso muscles for 15–20 s, then returning to a normal state for 20–30 s (Ost & Sterner, 1987). This cycle is repeated five times. The aim of this training was to see a rise in blood pressure resulting from the muscle-tension technique.

On her own initiative, P practiced the applied muscle-tension technique (Ost & Sterner, 1987) three to five times per day and assessed practice sessions using a blood pressure monitor she borrowed. She recorded systolic blood pressure and diastolic blood pressure during one practice session per day, taking baseline recordings after a resting period of five minutes prior to applied muscle tension and taking recordings at one minute during applied muscle tension and at three minutes directly after five muscle-tension cycles. There was an average increase of 8 mmHg (the usual measure of blood pressure) for systolic blood pressure *during* applied muscle tension and an average increase of 4 mmHg for systolic blood pressure *after* applied muscle tension. Diastolic blood pressure remained constant during and after applied muscle tension.

Following the suggestions of Miltenberger (2011), P developed a fear hierarchy using a range of situations associated with blood donation and successively rated each level with a baseline SUDS (subjective units of distress scale) score from 0 (no fear) to 100 (extreme fear). See Table 1 for the hierarchy items and baseline ratings. Following the suggestions of M, P also recorded SUDS ratings directly after each 8-min trial of gradual exposure to determine when a substantial drop indicated that it was time to move on to a successive level.

**Table 1.** Fear hierarchy with baseline, post-treatment, and follow-up SUDS ratings.

Hierarchy item	SUDS		
	Baseline	3 months	17 months
(1) Read blood-donation procedures online and in print	10	0	0
(2) Speak to blood donors about the process of blood donation and their experiences	20	0	0
(3) Speak to the blood donor center staff about blood-donation procedures	30	0	0
(4) Sit in the waiting room at the blood donor center	40	0	0
(5) Complete the donor questionnaire and interview process to become a blood donor	50	0	0
(6) Observe the blood donor room at a distance of 8–10 m	60	0	0
(7) Observe the preparation procedure for blood donation, including all equipment used	65	0	0
(8) Experience the preparation procedure for blood donation	70	20	20
(9) Observe a full blood donation	80	25	0
(10) Give a blood test sample	90	30	20
(11) Give a full blood donation	100	30	40

Before beginning exposure trials, P viewed a video of the successful treatment of a woman with snake phobia by Dr Lars-Goran Ost (Wadsworth, 2005). She also contacted family members, friends, and staff members at the local blood-donation center to inform them of her goal of overcoming her phobia. The staff members were helpful and praising; her friends and family congratulated her each time she informed them of progress.

After a week of applied-tension practice that totaled approximately three hours, P began arranging her own gradual exposure during in vivo exposure trials. She used applied muscle tension in all trials where she felt the onset of symptoms indicating a drop in blood pressure. She praised herself after every successful 8 min trial, and after each successful completion of a level, she informed a family member and sometimes a blood donor staff member of her completion. For social support at high levels of the hierarchy, P talked with family members or friends after trials. For support at trials at the highest levels of the hierarchy, she talked with family members or friends immediately before each trial. After each successful completion of levels six to nine, she purchased a new CD to listen to directly after the trials. P also began volunteering at the local blood-donation center.

P found it very time-consuming to perform trials in actual feared situations, so she changed to a combination of *in vivo* and imaginal exposure, an exposure option described by Miltenberger (2011), in which a person vividly imagines being in the feared situation. She used *in vivo* exposure in every initial trial and then recorded details of the trial to assist in imagining the feared situation in the following imaginal-exposure trials. This use of exposure in imagery allowed her to perform multiple trials at home and to minimize time constraints.

The approximate time P invested in the project included 2 h for creating the fear hierarchy, 0.25 h for viewing the phobia-treatment video, 4.5 h of initial practice of applied tension, 6.5 h of exposure trials, 6 h obtaining social support, and 0.75 h total discussing the project with M. That added up to 20 h.

## 3. Results

### 3.1. Initial results

P moved gradually through the hierarchy levels until she reached the highest level, donating blood, three months later. Along the way, she gave a small amount of blood for a medical test. The first donation of blood for the use of others did not go smoothly. The nurse was unable to find a vein in the chosen arm, possibly because of low blood pressure, so she switched to the other arm and successfully withdrew an amount for donation. The nurse took a photo during the donation for P to use in future exposure sessions. At that point, P no longer had any fear of blood withdrawal for medical tests. Table 1 shows that P's SUDS ratings were much lower than that at baseline. During three months of treatment, P completed about three volunteer shifts at the donation center, working immediately next to the glassed-in donation area.

### 3.2. Long-term follow-up

Over the next 14 months, P attempted to donate blood three more times. The first of these attempts ended prematurely due to uncertainty about whether P's iron level was high enough for a donation. She did, however, at that visit give blood for testing by both a finger prick and a venipuncture. In the subsequent two attempts, P donated blood. At 17 months after she began self-exposure, her SUDS ratings for the hierarchy levels were much lower than that at baseline (Table 1), and she showed no avoidance of blood-related stimuli or activities and no fainting. At that point, she continued to use muscle tension to maintain her blood pressure and to avoid becoming light-headed while donating blood. During these 14 months, P volunteered about once a month at the blood-donation center.

## 4. Discussion

The results demonstrate, for the first time, that a person can overcome BII phobia through self-arranged sessions of applied-tension practice and gradual exposure. The phobic individual learned from a journal article (Ost & Sterner, 1987) how to use applied muscle tension to prevent fainting, created a fear hierarchy on the basis of written instructions in a behavior-modification textbook (Miltenberger, 2011), and then completed one exposure trial after another. Working on her own to overcome the phobia, she made slow but sure progress, reaching the highest level of her hierarchy, donating blood, three months after beginning exposure trials. Her maintenance of non-avoidance over the subsequent 14 months shows durability of benefits. The improvement eliminated risks inherent in the fear of giving blood for medical tests and allowed her to make possibly life-saving donations of blood for use by others.

The findings add to prior findings that applied-tension practice and gradual exposure arranged by a professional can end blood-injection-injury phobia (Ayala et al., 2009; Holly, Torbit & Ditto, 2012; Lillecreutz et al., 2010; Mednick & Claar, 2012; Ost & Sterner, 1987). Eliminating the professional from the tension-reduction and exposure sessions has the potential value of making the treatment available to many individuals who would not or could not find a qualified, affordable professional to arrange the sessions for them. The current results provide relatively long follow-up evidence of positive effects for self-exposure treatment for BII phobia, at 17 months from the start of treatment.

The phobic individual in this case had the advantages of being an undergraduate psychology student, having extensive written materials on phobia treatment, and consulting a psychologist before she began work on ending her phobia. The written instructional materials helped her adjust the exposure trials by including imaginal exposure so that she could perform trials more frequently. Her education as a psychology undergraduate helped her understand the psychological principles she applied. P did not have the advantage of time pressure, which might occur if a person were paying a large sum of money for each treatment session. Possibly as a result of the lack of time pressure and the lack of direct therapist involvement in exposure trials, she took three months to reach her goal of donating blood. Her spreading out of exposure trials over substantial time may have decreased the value of prior exposure trials, making it harder for her to progress. One implication of this analysis is that it might be helpful to encourage phobic individuals using self-exposure to work consistently on exposure trials towards a near target date for achieving the ultimate goal.

The results are limited in that they come from only one person. It is unclear to what extent they might generalize to others with BII phobia. The main value of the findings is that they demonstrate the *possibility* of adults with BII phobia successfully arranging their own practice of applied muscle tension and their own exposure sessions.

It would be useful for mental health professionals to test similar methods with other individuals who have a specific phobia of some sort so that we can start to determine which phobic individuals can successfully use self-arranged gradual exposure to overcome a phobia. It could be that by providing minimal assistance of the sort that could be done via the Internet, psychologists can help a significant number of phobic individuals arrange their own exposure trials as part of a low-cost, accessible, and convenient alternative to psychologist-arranged exposure sessions. Examples of self-arranged exposure like the present one may provide useful models (Couch, Han, Robinson, & Komesaroff, 2014) for others with BII phobia.
